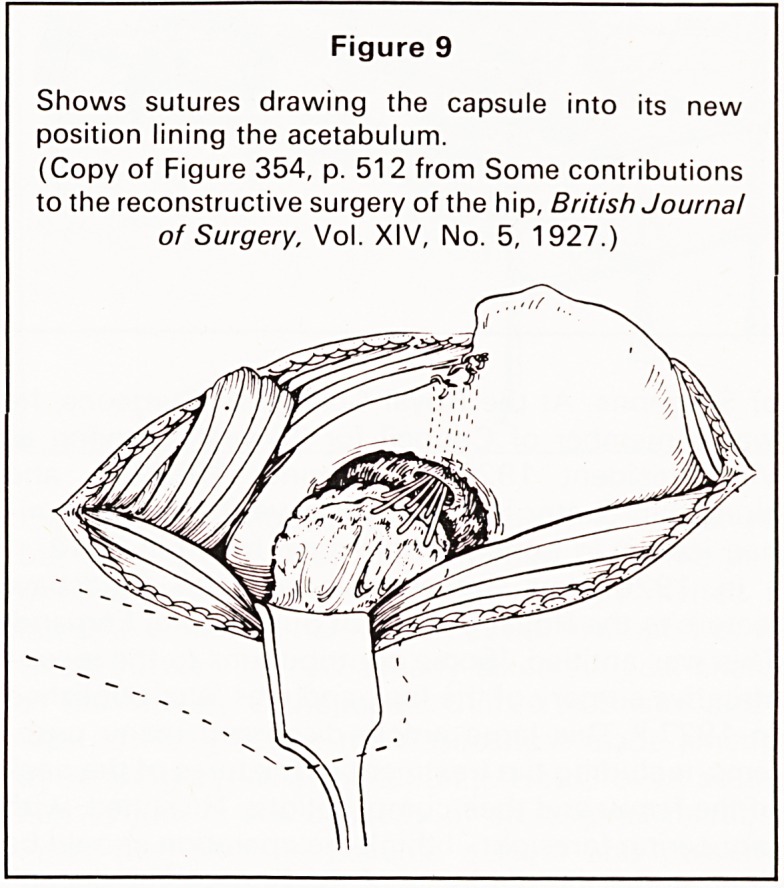# Ernest William Hey Groves and His Contributions to Orthopaedic Surgery

**Published:** 1983-07

**Authors:** Anthony H. C. Ratliff

**Affiliations:** Consultant Orthopaedic Surgeon, Bristol Royal Infirmary and Winford Orthopaedic Hospital

**Keywords:** Orthopaedics, history, Transplantation, Arthroplasty, Ligaments, Hip dislocation, Biography


### Ernest William Hey Groves and his Contributions to Orthopaedic Surgery

**Anthony H. C. Ratliff Ch.M., F.R.C.S.**
Consultant Orthopaedic Surgeon, Bristol Royal Infirmary and Winford Orthopaedic Hospital

*Keywords*: Orthopaedics, history; Transplantation; Arthroplasty; Ligaments; Hip dislocation; Biography

Ernest William Hey Groves was the son of an English civil engineer and was born in India on 20th June 1872. At the age of three, when his father retired, the family settled in Bristol. He inherited an aptitude for mechanics and took a B.Sc. in engineering. In London, while pursuing his science course, he saw an advertisement for a scholarship at St. Bartholomew's Hospital, London, which included the exact subjects which he was studying in his science degree. He entered for this scholarship as he said 'for a lark' and was successful. Neither his parents, nor his uncle with whom he lived, gave him an allowance, so he earned his maintenance by teaching biology. He later became a superb surgical lecturer. He qualified M.R.C.S., L.R.C.P. in 1895 and was a house surgeon in gynaecology for only 6 months before visiting Germany for one term as a student of physiology at the University of Tübingen. During this time he obtained a basic knowledge of the German language which proved invaluable to him in later years when he translated Böhler's classical monographs.

After qualification, his twin aims were to earn a living and to leave the city life which he had led in London for 5 years. He settled in a village in Somerset but after 2 years, realised that this country practice was too isolated for his personality and that he desired a more active medical life. However, during the time in Somerset, he completed his M.B.B.S. and later obtained the M.D. degree. Hey Groves moved to a busy practice at Kingswood in Bristol. One day he was consulted by a lady who complained of lumbar backache. He discovered that she had an ovarian cyst impacted in her pelvis. Delighted at receiving a definite diagnosis, the lady insisted that Hey Groves should remove the cyst himself. He had no nursing home or hospital facilities at that time and indeed, possessed no surgical instruments! He improvised a room at his home and with the help of his wife, who had been a nurse at St. Bartholomew's Hospital, the operation was successfully accomplished. This remarkable achievement earned Hey Groves a reputation and he then ran his own nursing home and with no surgical training, removed appendices, prostates and gall bladders. He use to say that the first gastroenterostomy he ever saw was one performed by himself. Such was his growing reputation that he was appointed to the surgical staff of the Bristol General Hospital in 1903. He obtained his Fellowship in 1905 and an M.S. with Gold Medal in the same year.

Paper read to the Royal College of Surgeons of England on 30th September, 1981, and to the Bristol Medico-Chirurgical Society in November 1981.

For four years he retained a share in the Kingswood practice, cycling there each morning and returning each afternoon to the Bristol General Hospital where he was consulting surgeon in charge of outpatients. During this early part of his career it was said, perhaps unkindly, that 'butcher Groves lured women into his home, operated upon them and would not remove their stitches until they had paid their money'.

Then came the really productive years when he became interested in research. He could not do any experimental work in Bristol so twice a week he travelled to University College Hospital in London to experiment on the union of fractures in cats and rabbits. He was fascinated by the operative treatment of fractures and in 1914 he gained a Hunterian Professorship on this subject. He demonstrated the need for external fixation in the treatment of fractures and showed that osteitis could occur, even with plating and that non-union could follow internal fixation (Figure 1). Experimental observations on the operative treatment of fractures were studied for a number of years and discussed in great detail, with many illustrations, in the *British Journal of Surgery.*[1]

In 1916 he obtained the Jacksonian Prize of the Royal College of Surgeons of England on, 'Methods and results of transplantation of bone in repair of defects caused by injury or disease'. He wrote a textbook entitled, *On Modern Methods of Treating Fractures* first published in 1916 and in the second edition (1921) he records his many experimental observations on bone grafting, having first described with characteristic clarity, the contributions of Ollier, Barth, Axhausen and MacEwen. He considered that the most ideal graft was a piece of living bone used in its entire thickness and that the success of this graft depended largely on the extent of its contact

**Figure 1**

*Left*: Tibia of cat treated by double transfixion, 21 days after fracture, showing perfect position and good union.
(Copy of Figure 123. p. 216 On Modern Methods of Treating Fractures, E. W. Hey Groves, 1921, John Wright & Sons Ltd.)
*Centre*: Femur of cat 6 weeks after plating, showing proliferative osteitis.
(Copy of Figure 162, p. 239 On Modern Methods of Treating Fractures, E. W. Hey Groves, 1921, John Wright & Sons Ltd.)
*Right*: Tibia of cat showing no callus and non-union 6 weeks after plating and application of split pins.
(Copy of Figure 53d. p. 98 On Modern Methods of Treating Fractures. E. W. Hey Groves, 1916, John Wright & Sons Ltd.)

**Figure 2**

The fixation of a sub-trochanteric fracture using bone peg.
(Copy of Figure 269. p. 358 On Modern Methods of Treating Fractures, E. W. Hey Groves, 1921, John Wright & Sons Ltd.)

**Figure 3**

Intra-medullary fixation of a fracture.
(Copy of Figure 271, p. 360 On Modern Methods of Treating Fractures, E. W. Hey Groves. 1921. John Wright & Sons Ltd.)

Modern Methods of Treating Fractures with living bone. Accuracy of apposition and firmness of fixation was of paramount importance in the repair of the defects of long bones by grafting.[2] He developed the use of bone pegs in, for example, the fixation of a subtrochanteric fracture (Figure 2). The principles of treatment of fractures of the shaft of the femur, later ascribed to Küntscher, are very clearly illustrated in this book, although Hey Groves believed in fixation of the fracture with a bone peg rather than an intra-medullary nail (Figure 3). He stated that 'delay in union is a possible, though exceptional result of perfect operative fixation of a fracture'.[3] It is remarkable that this was written in

1916 and yet the need for rigid internal fixation in the production of union of fractures is still a highly debatable and controversial subject.[4]

In 1908 Hey Groves had published a *Synopsis of Surgery,* based on his own fellowship notes. There were eventually 11 editions of this book and its considerable success prompted thoughts concerning the regular publication of a surgical journal. He was horrified that the country which had produced outstanding contributions from Hunter and Lister did not have its own Journal of Surgery and he became a founder member of the *British Journal of Surgery,* remaining its editor for 27 years. He aimed to make it the leading surgical journal in the world. He was convinced that a successful journal must have the active support of one or more surgeons from every teaching centre in Great Britain. He said: 'we had to make sure that the active minds of the provinces should be joined to those in the Metropolis so as to avoid the dangers of a publication in London where the rivalry of the great schools has often prevented concerted effort'.[5] The first edition appeared in July 1913. Hey Groves did an enormous amount of tedious editorial work, including the translation of scientific articles from France and Germany and after 20 years' service, he was presented with a silver salver by Lord Moynihan under whose guidance and affection he had worked. In October 1914, the Editorial Committee seriously debated whether it was worthwhile continuing a surgical journal at that crisis in history but fortunately this was not agreed and special articles dealing with military surgery were accepted and these established the reputation of the Journal and proved to be its salvation.

The war gave Hey Groves a great impetus to pursue the surgery of bones and joints. First, there was a great wealth of material (he wrote a small book on Gunshot Injuries to Bones) and second, the organisation of orthopaedic work brought him into contact with Sir Robert Jones and 'under the sway of his gentle but compelling genius'. Hey Groves later said of the 1914–18 war, 'apart from the horrors and tragedy, they were gloriously happy years'. He appreciated particularly the camaraderie of the profession; English, French, American and Canadian surgeons all working together.

He described his own splint for the treatment of fractures of the lower limb, the principles of which are still in universal use today. Many do not realise that Hey Groves was the originator of the principle of the Stryker frame for the treatment of bullet wounds of the spine and paraplegia (Figure 4). He wrapped the patients in this type of collapsible device and turned them over in exactly the same way as is advocated today.

On 28th November 1917, Hey Groves was one of that small group of surgeons who met for dinner at the Café Royal in London. As a result of this meeting, the British Orthopaedic Association was founded but Hey Groves was still regarded by some as a general surgeon and was not originally invited to become a member. However, at the invitation of Sir Robert Jones, he had already taken surgical charge of the Military Orthopaedic Centre in Bristol and it was not long before the Association made amends by sending a special invitation to Hey Groves, asking him to join in the capacity of an original member. From that time he became a loyal and powerful advocate of the cause of orthopaedic surgery.

**Figure 4**

Revolving spinal bed. In the upper figure the frame is open. In the lower figure the frame is closed and in this position a patient can be turned over.
(Copy of Figure 6. p. 15 from A surgical adventure, *Bristol Medico-Chirurgical Journal*, Spring 1933.)

Hey Groves wrote two classical articles on injuries of the cruciate ligaments of the knee joint and their repair; one in 1917[6] and the other in 1919[7] and an illustration from the latter article shows how he pulled the tendons through into the front of the knee joint, threaded them through the medial femoral condyle and attached them to the medial ligament, thus repairing the posterior cruciate ligament (Figure 5). At the end of this article he wrote with characteristic integrity; 'I am sure that I could do much better with the earlier cases if I had to do them again'.

He was appointed Professor of Surgery in Bristol in 1922 and quickly became a national and international figure in surgery. In 1928–9 he was President of the British Orthopaedic Association and in the following year was President of the Association

**Figure 5**

Repair of the posterior 'crucial' ligament.
*Left*: A tunnel (A) is being drilled through the internal condyle of the femur. The tendons of the gracilis and semitendinosus (B and C) have been brought into the joint from the back.
*Right*: The two tendons have been drawn through the tunnel in the internal femoral condyle and then turned downwards to supplement the internal lateral ligament.
(Copy of Figures 426 and 427, p. 513, *British Journal of Surgery*, 1919–20, Vol. 7.)

of Surgeons. At the Royal College of Surgeons, he was a member of Council for 23 years, serving as Vice-President 1928–9, examiner 1928–34, and Hunterian Orator in 1930. He gave the first Moynihan lecture in honour of his great friend in 1940.

In 1926 Hey Groves delivered the Bradshaw lecture to the Royal College of Surgeons of England. This was entitled, 'Some contributions to the reconstructive surgery of the hip', and was later published in 1927.[8] This large article discussed many problems, including the treatment of fractures of the neck of the femur and their complications. He stated, with pioneering foresight: 'I think the operation should be done under the guidance of the fluorescent screen'. He described an introducer, now well known, for insertion of a Smith–Petersen nail for fractures of the neck of the femur. This introducer has been said to make a very difficult operation simple. The treatment of ankylosis of the hip was also discussed and there follows a description of how to perform an arthroplasty of the hip through an antero-lateral exposure with the use of the capsule of the joint as an envelope for the end of the femur (Figure 6). He also illustrated one of the first arthroplasties of the hip using an ivory nail to replace the arthritic head (Figure 7).

In 1928, the famous *Robert Jones Birthday Volume* was published and in it Hey Groves wrote a chapter on Congenital dislocation of the hip joint. He commenced with the classical sentence: 'Congential dislocation of the hip is a deformity which is mysterious in its origin, insidious in its course and relentless

**Figure 6**

Arthroplasty of the hip, showing exposure of the neck of the femur by division of the capsule. Inset above, line of capsule division; below the neck of the femur enveloped in capsule.
(Copy of Figure 324, p. 500 from Some contributions to the reconstructive surgery of the hip, *British Journal of Surgery*, Vol. XIV, No. 55, 1927.)

in its final crippling results'.[9] He included diagrams of his operation for deepening the shallow acetabulum in the adolescent child which was later described and popularised by Colonna. The voluminous capsule is dissected free, divided and the cut edges sutured over the head of the femur, thus preventing fibrous union. The acetabulum is then deepened, the sutures passed through its base and an arthroplasty of the hip performed with the head of the femur covered by its own capsule (Figures 8 and 9).

Ernest Hey Groves was a great pioneer and original thinker and played a considerable part in the development of orthopaedic and traumatic surgery as we know it today. He gave a Presidential Address to the Medico-Chirurgical Society of Bristol in 1932 entitled, 'A Surgical Adventure' and in it he said 'Life must always be somewhat of an adventure, a mysterious reaction of the personality to the environment'.[10] His judgement was sound; his technique

**Figure 7**

Arthroplasty by the use of an ivory nail replacing the head of the femur.
(Copy of Figure 325, p. 501 from Some contributions to the reconstructive surgery of the hip, *British Journal of Surgery*, Vol. XIV, No. 55, 1927.)

**Figure 8**

Drawing of operation for transplanting capsule inside the acetabulum. A. New acetabulum. B. Cut edges of capsule being sewn together by sutures which are left long and then taken through a hole in the floor of the acetabulum.
(Copy of Figure 353, p. 512 from 'Some contributions to the reconstructive surgery of the hip, *British Journal of Surgery,* Vol. XIV, No. 55, 1927.)

**Figure 9**

Shows sutures drawing the capsule into its new position lining the acetabulum.
(Copy of Figure 354, p. 512 from Some contributions to the reconstructive surgery of the hip, *British Journal of Surgery,* Vol. XIV, No. 5, 1927.)

admirable. No-one could be bolder when boldness was required, yet he could be as cautiously conservative as anyone, upon occasion. He was a man of considerable literary, linguistic and surgical ability. He died in 1944 aged 72 years, a great pupil of Sir Robert Jones.

**ACKNOWLEDGEMENTS**

The author would like to express his appreciation to many colleagues in Bristol for advice, to the Department of Medical Illustration at the Bristol Royal Infirmary for copying and updating some of the illustrations from their original source, and especially to Mrs S. Collins for all her industry and expert secretarial help.

### REFERENCES

1. HEY GROVES E. W. (1913–14) An experimental study of the operative treatment of fractures. *Br. J. Surg.* **1**, 438–501.
2. HEY GROVES E. W. (1921) On Modern Methods of Treating Fractures. Bristol, John Wright & Sons Ltd. p. 247.
3. HEY GROVES E. W. On Modern Methods of Treating Fractures. Bristol, John Wright & Sons Ltd., p. 158.
4. MULLER M. E. ALLGÖWER M. and WILLENEGGER H. (1965) Technique of Internal Fixation of Fractures. Berlin, Heidelberg, New York, Springer-Verlag.
5. HEY GROVES E. W. (1933). A surgical adventure: an autobiographical sketch. *Bristol Medico-Chirurgical Journal*, **9**, Spring 1–22.
6. HEY GROVES E. W. (1917). Operation for the repair of the crucial ligaments. *Lancet.* **11**, 674–675.
7. HEY GROVES E. W. (1919). The crucial ligaments of the knee-joint: their function, rupture and the operative treatment of the same. *Br. J. Surg.* **7**, 505–515.
8. HEY GROVES E. W. (1927). Some contributions to the reconstructive surgery of the hip. *Br. J. Surg.* **14**, 486–517.
9. HEY GROVES E. W. (1928). The treatment of congenital dislocation of the hip joint, with special reference to open operative reduction. In The Robert Jones Birthday Volume—A Collection of Surgical Essays. Oxford University Press, pp. 73–96.
10. HEY GROVES E. W. (1933). A surgical adventure: an autobiographical sketch. *Bristol Medico-Chirurgical Journal*, Spring 2.

## Figures and Tables

**Figure 1 f1:**
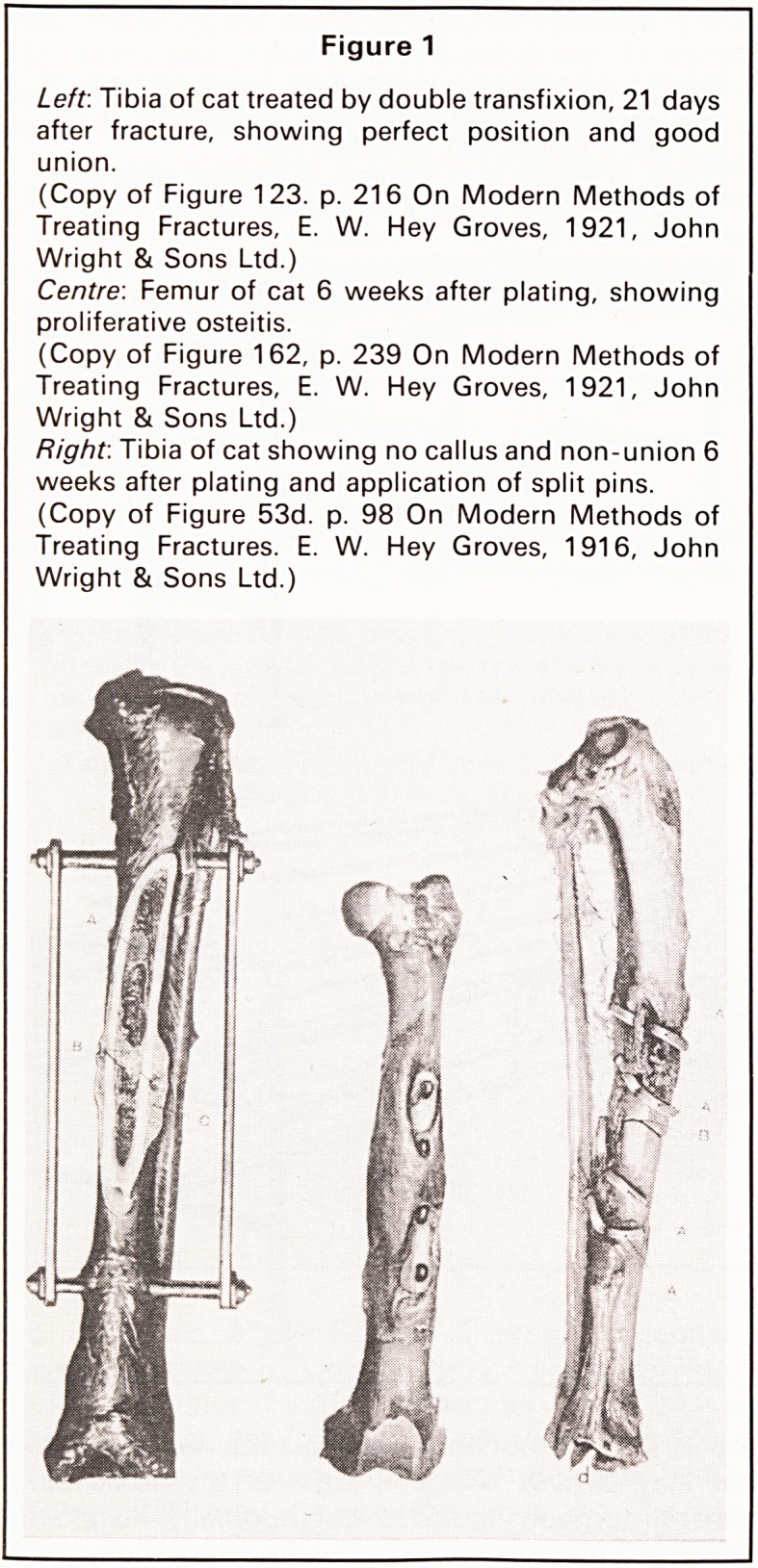


**Figure 2 f2:**
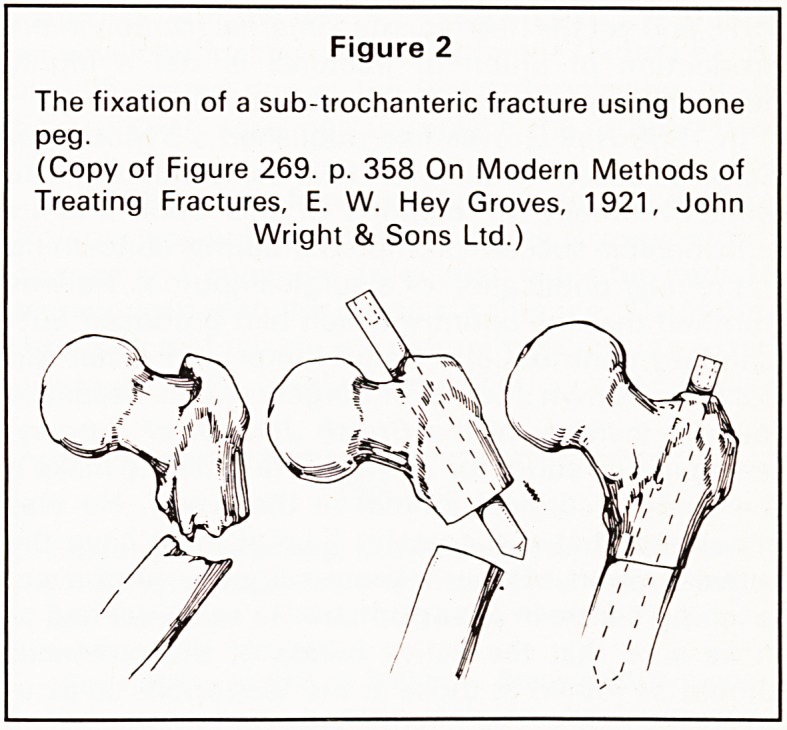


**Figure 3 f3:**
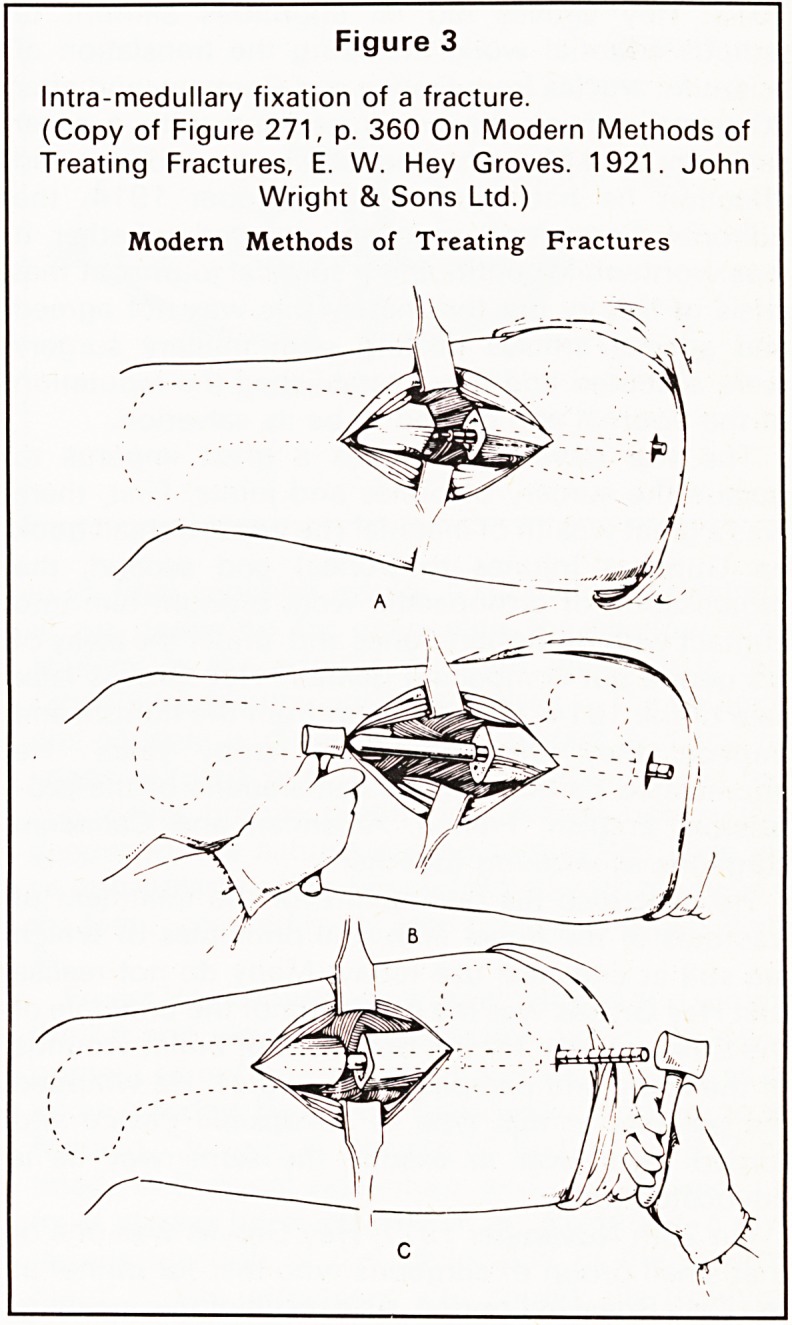


**Figure 4 f4:**
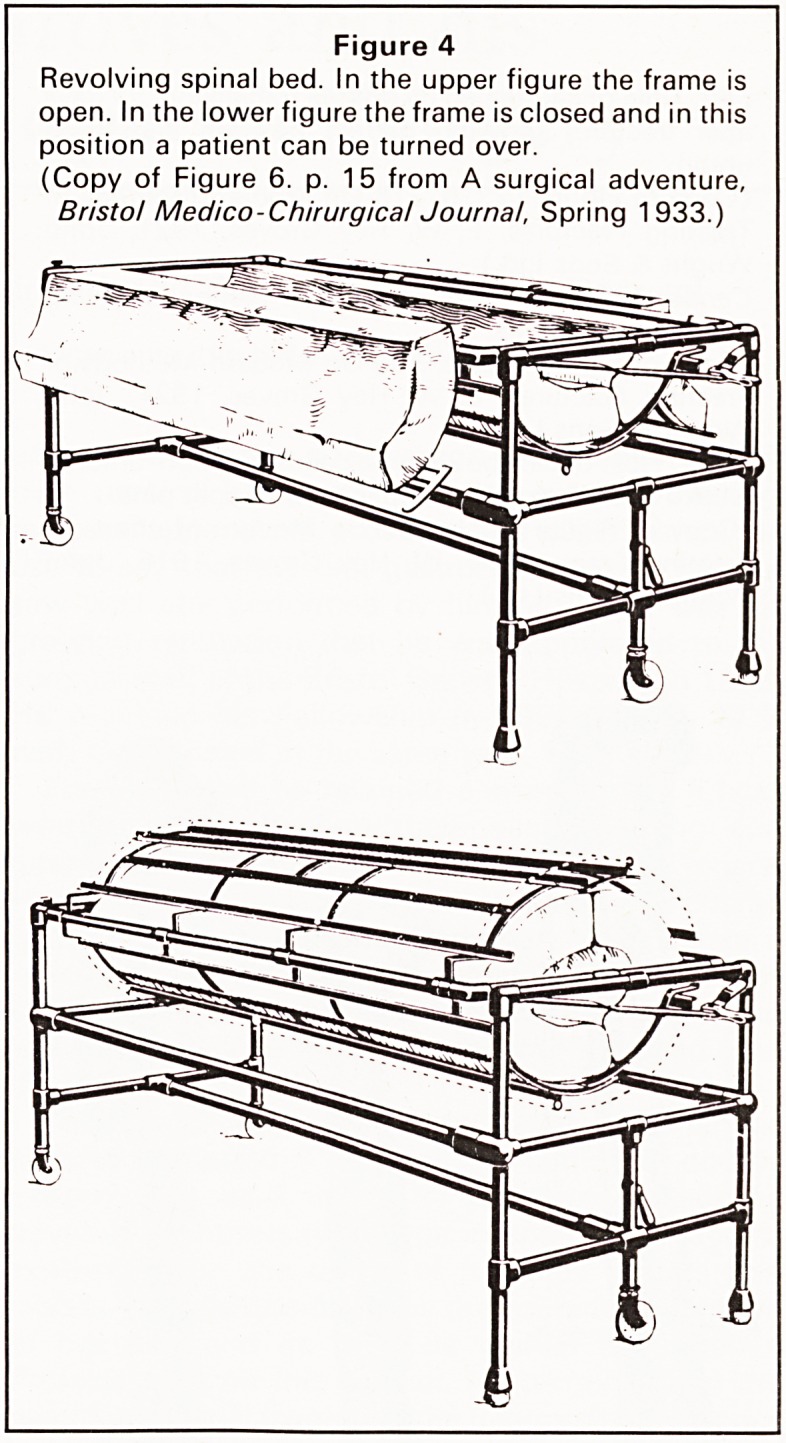


**Figure 5 f5:**
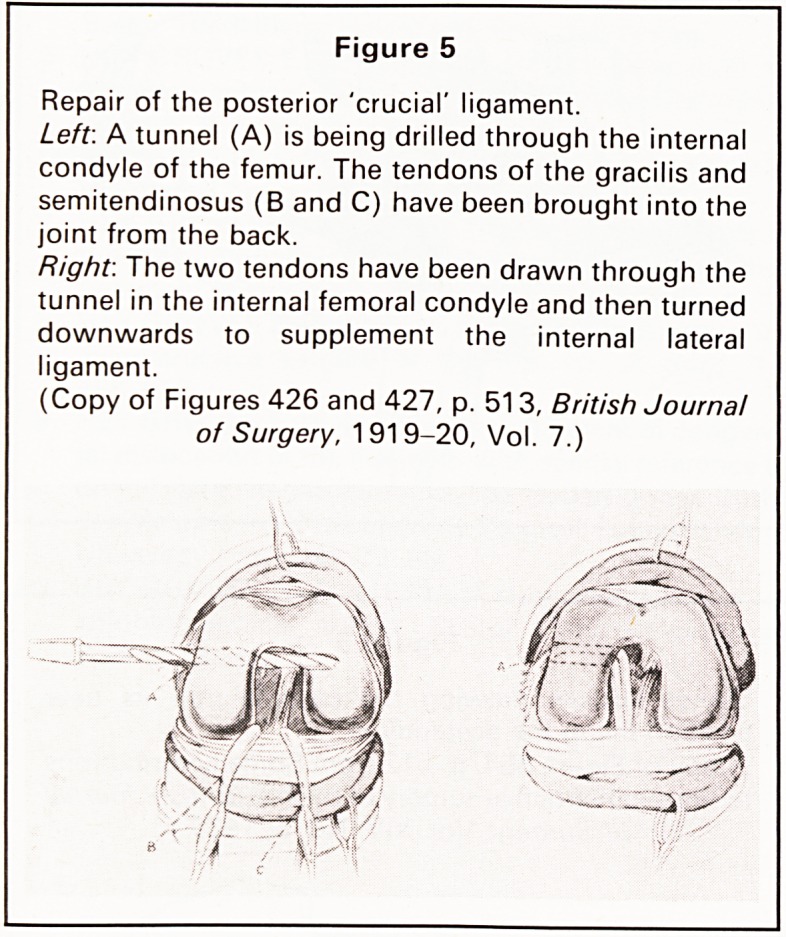


**Figure 6 f6:**
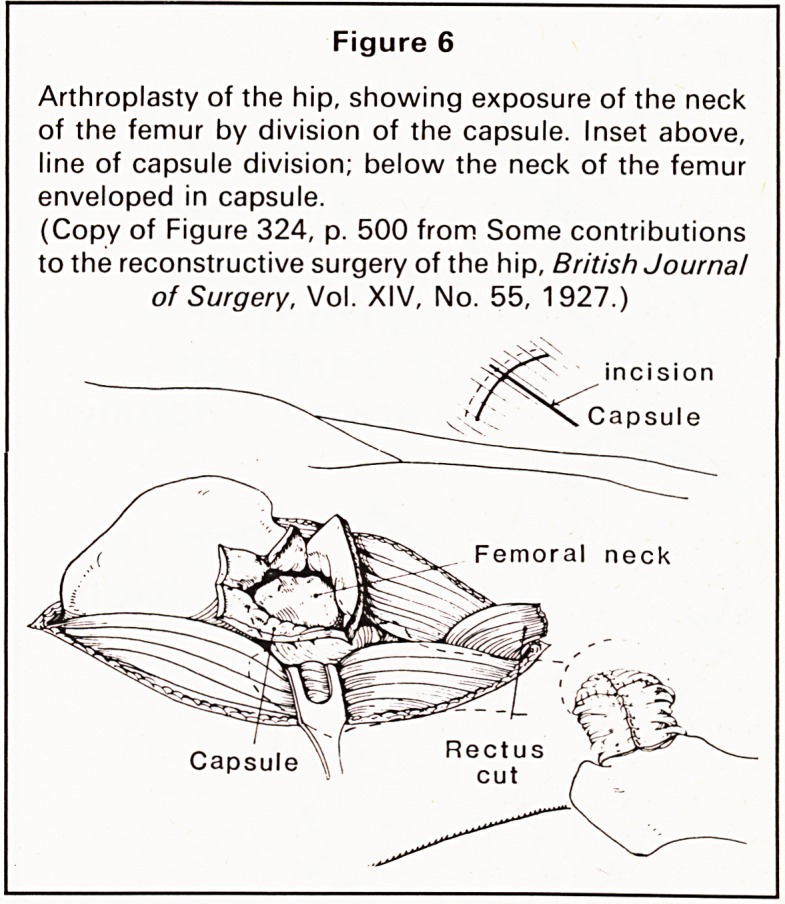


**Figure 7 f7:**
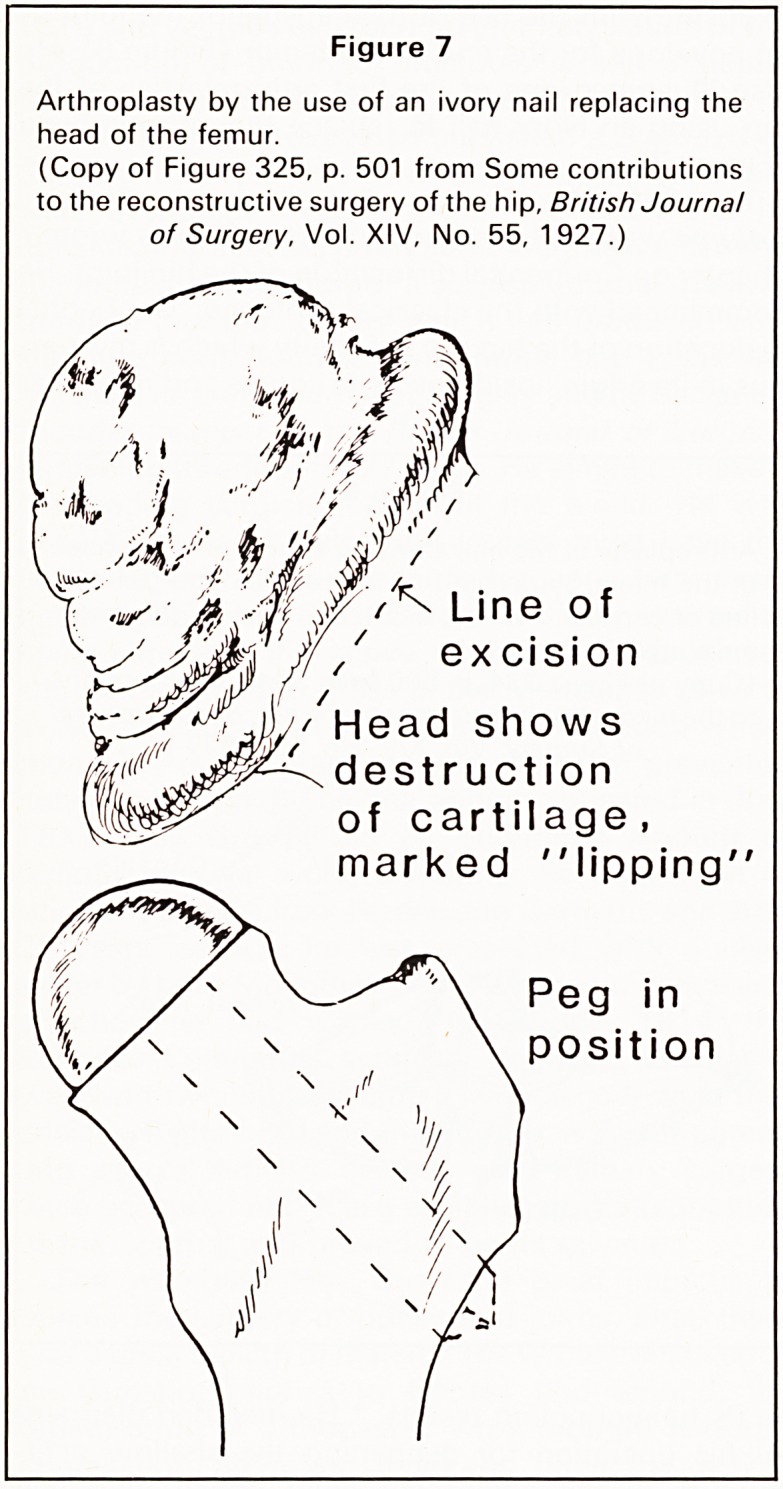


**Figure 8 f8:**
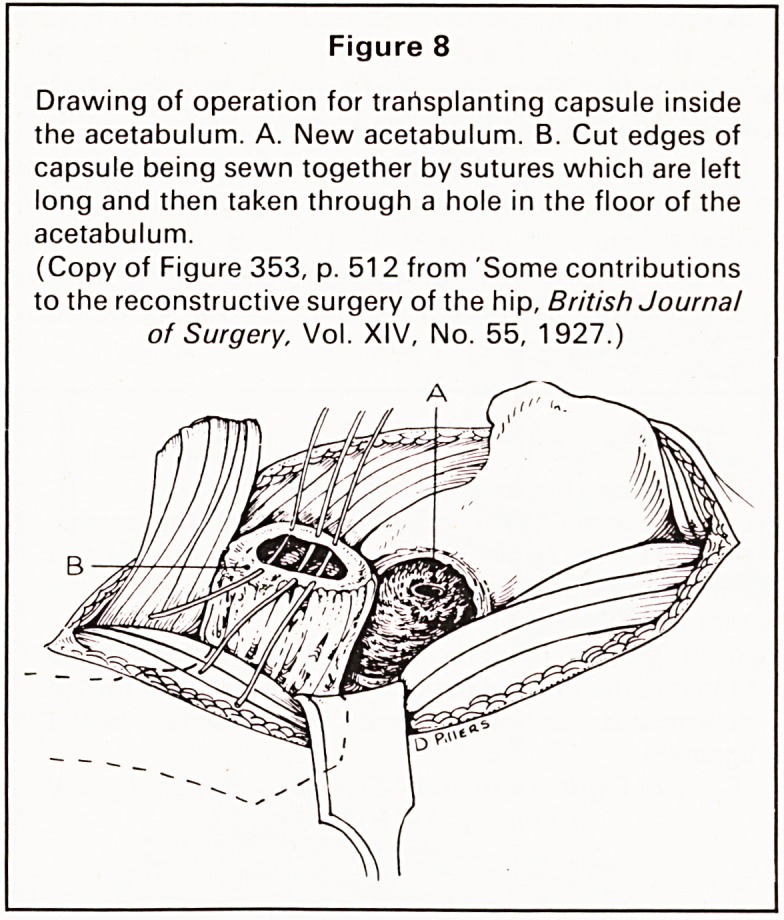


**Figure 9 f9:**